# Insulin-Like Growth Factor I Does Not Drive New Bone Formation in Experimental Arthritis

**DOI:** 10.1371/journal.pone.0163632

**Published:** 2016-10-03

**Authors:** Melissa N. van Tok, Nataliya G. Yeremenko, Christine A. Teitsma, Barbara E. Kream, Véronique L. Knaup, Rik J. Lories, Dominique L. Baeten, Leonie M. van Duivenvoorde

**Affiliations:** 1 Amsterdam Rheumatology and Immunology Center, Department of Clinical Immunology and Rheumatology, Academic Medical Center / University of Amsterdam, Amsterdam, The Netherlands; 2 Department of Experimental Immunology, Academic Medical Center / University of Amsterdam, Amsterdam, The Netherlands; 3 Department of Medicine, Uconn Health, Farmington, CT, United States of America; 4 Laboratory of Tissue Homeostasis and Disease, Skeletal Biology and Engineering Research Center, KU Leuven, Leuven, Belgium; 5 Division of Rheumatology, University Hospitals Leuven, Leuven, Belgium; Universite de Nantes, FRANCE

## Abstract

**Introduction:**

Insulin like growth factor (IGF)-I can act on a variety of cells involved in cartilage and bone repair, yet IGF-I has not been studied extensively in the context of inflammatory arthritis. The objective of this study was to investigate whether IGF-I overexpression in the osteoblast lineage could lead to increased reparative or pathological bone formation in rheumatoid arthritis and/or spondyloarthritis respectively.

**Methods:**

Mice overexpressing IGF-I in the osteoblast lineage (Ob-IGF-I^+/-^) line 324–7 were studied during collagen induced arthritis and in the DBA/1 aging model for ankylosing enthesitis. Mice were scored clinically and peripheral joints were analysed histologically for the presence of hypertrophic chondrocytes and osteocalcin positive osteoblasts.

**Results:**

90–100% of the mice developed CIA with no differences between the Ob-IGF-I^+/-^ and non-transgenic littermates. Histological analysis revealed similar levels of hypertrophic chondrocytes and osteocalcin positive osteoblasts in the ankle joints. In the DBA/1 aging model for ankylosing enthesitis 60% of the mice in both groups had a clinical score 1<. Severity was similar between both groups. Histological analysis revealed the presence of hypertrophic chondrocytes and osteocalcin positive osteoblasts in the toes in equal levels.

**Conclusion:**

Overexpression of IGF-I in the osteoblast lineage does not contribute to an increase in repair of erosions or syndesmophyte formation in mouse models for destructive and remodeling arthritis.

## Introduction

Rheumatoid arthritis (RA) and spondyloarthritis (SpA) are the two most frequent forms of chronic inflammatory arthritis [1]. Both diseases are not only characterized by joint inflammation but also by marked structural damage to bone, cartilage, and soft tissues leading ultimately to loss of function of affected joints. The phenotype of the structural damage, however, is strikingly different between RA and SpA. RA is characterized by progressive destruction of cartilage and bone in the virtual absence of any repair and new bone formation, even when inflammation is completely controlled. Whereas cartilage and bone destruction are also important features of SpA, the structural phenotype is dominated by pathological new bone formation eventually leading to complete ankylosis of the axial and/or peripheral joints [2]. In physiological conditions, bone resorption and new bone formation are balanced and tightly coupled processes. The molecular mechanisms of excessive bone erosion in inflammatory arthritis are relatively well understood, with a central role for TNF and the receptor activator of nuclear factor kappa-B ligand (RANKL) in the maturation and activation of osteoclasts [3]. Accordingly, blocking TNF and/or RANKL almost completely abrogates progression of bone erosions in experimental and human inflammatory arthritis [3]. Moreover, in hTNF transgenic mice, TNF is able to suppress new bone formation during chronic inflammation by up regulating DKK-1, an inhibitor of the wingless protein (Wnt) pathway [4]. These observations, however, fail to explain why bone repair is not occurring after inhibiting inflammation in RA and why new bone formation does occur during active inflammation in SpA [5–7]. A better understanding of which molecular pathways are involved in new bone formation in the arthritic joints may help us to decipher and eventually modulate the structural phenotype of RA and SpA.

The most studied molecular pathways in osteogenesis during inflammatory arthritis are the Wnt pathway [8] and the bone morphogenic protein (BMP) pathway [9], both of which are important in driving chondro- and osteogenic cell differentiation. A third pathway, which has not been studied extensively in the context of arthritis, is the insulin-like growth factor (IGF)-I pathway [10, 11]. IGF-I is a growth factor acting on a variety of target cells, including chondrocytes and osteoblasts [12]. IGF-I can drive mesenchymal cells towards chondrocyte differentiation [13], up regulate chondrocyte anabolism, and thereby enhance cartilage repair [14]. Moreover, IGF-I acts as chemoattractant for osteoblasts [15]. Its relevance of homeostatic bone turnover is evidenced by the fact that serum levels of IGF-I correlate with bone formation and bone resorption markers [16] and levels drop in aging individuals [10]. As to arthritic conditions, we showed previously that IGF-I is one of the major drivers of melanoma inhibitory activity (MIA) expression, a biomarker of cartilage anabolism, in arthritis [17]. Furthermore an IGF-I gene polymorphism was found to relate to low serum IGF-I levels and RA severity [18]. Collectively, these findings indicate that IGF-I could be an important molecular player controlling new bone formation in inflammatory arthritis. Therefore, this study aimed to investigate the potential role of IGF-I in repair of erosions and/or formation of syndesmophytes in experimental models of RA and SpA, respectively, taking advantage of the Ob-IGF-I^+/-^ mice [19]. These animals have osteoblast-lineage specific IGF-I overexpression under the rat collagen type 1 alpha I promoter and display an increase in bone density and enhanced growth. Local inflammation plays a center role in inflammatory arthritis, by studying IGF-I overexpression under the osteoblast promotor, in this paper we specifically focus on inflammation induced pathology in the joint at the local site of erosions in RA and pathological syndesmophyte formation in SpA. We hypothesized that overexpression of IGF-I in the osteoblast lineage could lead to an increase repair of erosions, typical for RA-like disease and/or to an increase in pathology caused by syndesmophyte formation suggestive of SpA-like disease.

## Materials and Methods

Animal experiments were performed in accordance with the animal ethical committee Academic Medical Center/University of Amsterdam, Amsterdam, the Netherlands. Permit numbers DRI-102402 and DRI-102702. Mice were euthanised using compressed CO2 gas, first in combination with O2 (70%/30% ratio) followed by 100% CO2. This study was approved by the animal ethical committee Academic Medical Center/University of Amsterdam, Amsterdam, the Netherlands.

### Mice

Ob-IGF-I^+/-^ mice (324–7) [19], kindly provided by Dr. B. Kream from the University of Connecticut Health Center, were backcrossed for at least 10 generations on the genetic DBA/1OlaHsd background. During all animal experiments efforts were made to minimize suffering.

### Collagen induced arthritis

CIA was induced in Ob-IGF-I^+/-^transgenic mice and non-transgenic littermates (n = 9 males per group) as described [20]. Mice were followed up for 60 days and sacrificed for histological analysis. One wildtype was taken out of the study at day 52 due to reaching the humane endpoint for arthritis severity; last observation was carried forward.

### Ankylosing enthesitis DBA/1 aging model

Ob-IGF-I^+/-^transgenic mice and non-transgenic littermates (n = 15 males per group) from various litters were mixed and caged together at 10 weeks of age in standard cages (6 mice/cage), mice were scored twice a week as described before [21]. Mice were followed-up for 16 weeks and sacrificed for histological analysis. Three mice were taken out of the study after 4–7 weeks due to severe bite wounds; last observation was carried forward.

### Histology

From the 9 mice per group in the CIA experiment both ankles (n = 18) were used for histology, 6 out of 18 mice per group in the ankylosing enthesitis model had clinical disease and toes from both feet were selected for histology (n = 12). Accordingly, bone tissues were fixed in 4% formalin, decalcified in EDTA or Osteosoft (Merck) and paraffin-embedded. 5 μm serial sections were cut and stained with Hematoxilin and Eosin, Safranin O (Sigma-Aaldrich) or Tartrate Resistant Acid Phosphatase (TRAP) according to manufacturer’s instructions (Sigma-Aaldrich) for ankle sections after CIA only. Ankle slides were semi-quantitatively scored for inflammation, destruction, proteoglycan loss, presence of hypertrophic chondrocytes as a measure for new bone formation and TRAP positive cells to indicate osteoclasts. Toes were scored for hypertrophic chondrocytes. Scoring was performed by two independent blinded observers (MvT, LvD) using a 0–3 scoring system (0 = no, 1 = mild, 2 = moderate 3 = severe pathology).

### Immunohistochemistry

Osteoblasts in ankle and toe sections were stained for osteocalcin, accordingly, antigen retrieval was performed with proteinase K at 37°C, followed by protein block and overnight incubation at 4°C with 1 μg/ml rabbit-anti-mouse polyclonal osteocalcin antibody (#ALC-210-333 Enzo life sciences) or 1 μg/ml rabbit Ig fractions isotype (DAKO). Slides were semi-quantitatively scored by two independent blinded observers (MvT, LvD) using a 0–3 scoring system.

### Statistical analysis

Mann-Whitney U tests were performed using Graph pad Prism 5 software, for clinical data area under the curve was calculated followed by a Mann-Whitney U test. P values below 0.05 were considered statistically significant.

## Results

### Overexpression of IGF-I in the osteoblast lineage does not lead to bone repair in CIA

**Fig 1:**
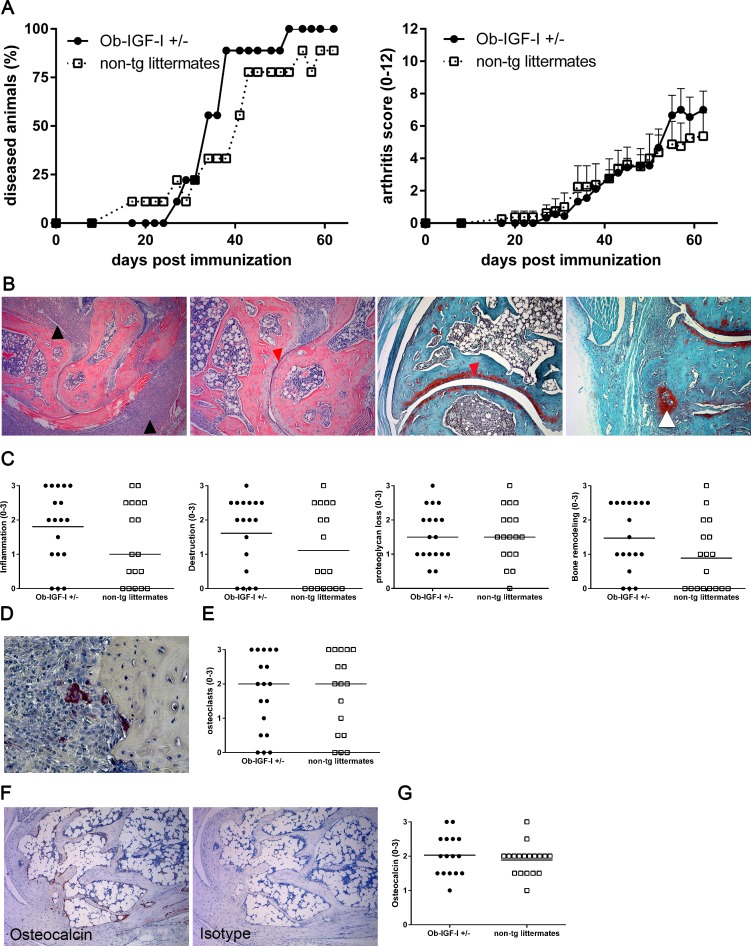
IGF-I overexpression does not lead to reparative bone remodeling in collagen induced arthritis. CIA was induced in Ob-IGF-I^+/-^ and controls (n = 9 per group). Arthritis incidence and severity were comparable (A). Severity is shown for diseased mice only. Data are mean ± SEM. Representative pictures of ankle joints show inflammation, destruction, proteoglycan loss and hypertrophic chondrocytes (magnification respectively 50x, 100x, 100x and 100x; black arrows: inflammation, red arrow: destruction/proteoglycan loss, white arrow: hypertrophic chondrocytes) (B) and quantification (C). TRAP positive osteoclasts detected in ankle sections (magnification 100x) (D) and quantification of osteoclasts (E) Osteocalcin and isotype stained ankle sections (magnification 100x) (F) and quantification of osteocalcin positive cells (G). Each data point represents one ankle, bars show median.

To study the effect of IGF-I overexpression in the osteoblast lineage on joint repair in destructive arthritis, CIA was induced in Ob-IGF-I^+/-^ mice and non-transgenic littermates. Disease incidence and development were similar, with an incidence of 90% and 100% in non-transgenic mice and Ob-IGF-I^+/-^ mice, respectively. Disease was equally severe (p = NS), arthritis development started around 14–21 days after immunization with a mean arthritis score of 5–7 per mouse at day 60. (Fig 1A). Histologically, inflammatory infiltrates were present in synovium, connective tissue, cartilage and bone in both groups. Joint destruction and erosions were present in both groups as indicated by the overall destruction and proteoglycan loss in the ankle sections (Fig 1B). Also the presence of hypertrophic chondrocytes in connective tissue could be observed in both groups (Fig 1B). Quantification of histological features indicated a similar degree of inflammation, cartilage and bone destruction, proteoglycan loss and hypertrophic chondrocytes suggestive for bone formation (p = NS) (Fig 1C). To confirm similar presence of erosions a TRAP staining for osteoclasts was performed. TRAP positive osteoclasts were mainly present near the site of pathology (Fig 1D), there were no differences in osteoclast numbers between the Ob-IGF-I^+/-^ mice and non-transgenic littermates (Fig 1E) To confirm the absent effect of IGF-I overexpression on the repair of erosions, we additionally stained osteocalcin positive osteoblasts in hind limb sections of all mice. As expected, osteocalcin positive osteoblast were mainly found near the periosteum; Isotype control staining was completely negative (Fig 1F). Quantification indicated similar numbers of osteocalcin positive cells in Ob-IGF-I^+/-^ mice and non-transgenic littermates (p = NS) (Fig 1G). Taken together, these data show that overexpression of IGF-I in the osteoblast lineage did not affect CIA, neither in terms of clinical disease and inflammation nor in terms of structural phenotype in general and repair of bone erosions in particular.

### Overexpression of IGF-I in the osteoblast lineage does not enhance pathological new bone formation in ankylosing enthesitis

**Fig 2:**
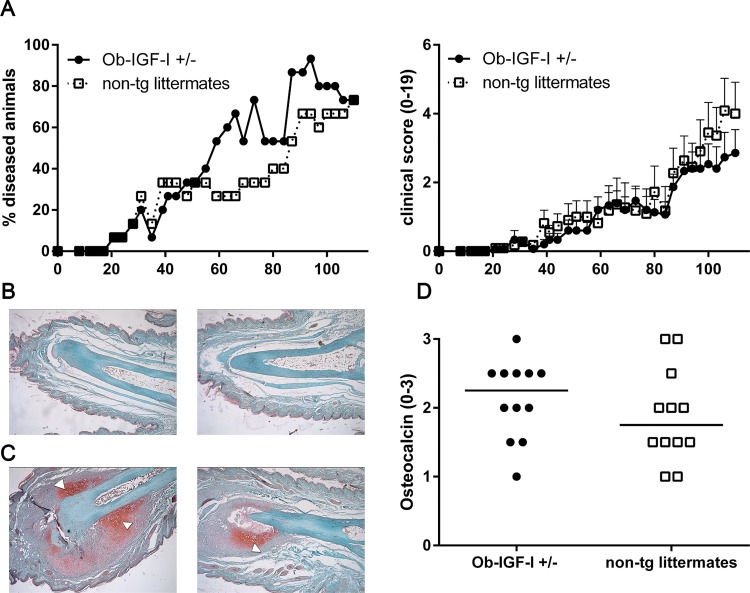
IGF-I overexpression in osteoblasts does not affect new bone formation in the DBA/1 aging model for ankylosing enthesitis. Aging male DBA/1 mice were followed up for 110 days and scored for spontaneous development of remodeling arthritis. Disease incidence and severity were comparable in both groups (A). Severity is shown for diseased mice only. Data are mean ± SEM. Safranin O staining revealed in both groups healthy toes (B) and toes with clear appearance of hypertrophic chondrocytes (C) (Magnification: 100x; white arrows point towards regions with hypertrophic chondrocytes). Quantification of osteocalcin positive osteoblasts revealed no differences (D).

As IGF-I did not appear to play a crucial role in the repair of bone eriosions in a model of destructive inflammatory arthritis, we next tested if IGF-I overexpression increases the formation of syndesmophytes in remodeling arthritis using the DBA/1 aging model for ankylosing enthesitis [22]. 10 week old Ob-IGF-I^+/-^ mice and non-transgenic littermates (n = 15/group) from various litters were mixed and housed together (6 mice/cage) [21]. Dactylitis and enthesitis started 20 days after mixed caging and slowly progressed to a disease incidence of 70% in both groups after 16 weeks of follow-up. Disease incidence and severity were comparable between groups (p = NS) (2A). To investigate the potential role for IGF-I on syndesmophyte formation, the presence of hypertrophic chondrocytes, detected by Safranin O staining, and osteocalcin positive osteoblasts, was analyzed histologically after 16 weeks of clinical follow-up. Hypertrophic chondrocytes were mainly found in the distal part of the toes surrounding the small joints and were present in both groups, two of the IGF-I transgenic mice had hypertrophic chondrocytes in the toes and three of the non-transgenic littermates (Fig 2B and 2C). Based on Safranin O positive staining six slides per group were selected for osteocalcin staining. Osteocalcin positive osteoblasts were found as expected near the periosteum (data not shown). Quantification of osteocalcin positive osteoblasts in Ob-IGF-I^+/-^ mice and non-transgenic littermates revealed similar levels of positivity (p = NS) (Fig 2D). Thus, IGF-I overexpression did not enhance syndesmophyte formation in the ankylosing enthesitis model.

## Discussion

In the present study we investigated the potential role of IGF-I in bone remodeling during chronic joint inflammation in mice. Mice with IGF-I overexpression in the osteoblast lineage show an increase in bone density under homeostatic conditions [19]. To test the hypothesis that IGF-I overexpression could also contribute to repair of erosions in destructive arthritis, we performed CIA experiments in mice overexpressing IGF-I in the osteoblast lineage. Our results, however, indicated no differences in either clinical disease parameters or structural phenotype between the Ob-IGF-I^+/-^ mice and non-transgenic littermates, indicating that local IGF-I overexpression, produced by activated osteoblasts, does not contribute to bone repair in destructive arthritis.

Although the clinical and histological observations were consistent in showing the absence of effect, the data should be interpreted with caution and do not allow to conclude that IGF-I is not important in destructive arthritis. Indeed, we mainly focused this study on repair of erosions and did not assess whether or not IGF-I overexpression protects from peri-articular osteoporosis, a prominent feature of destructive arthritis such as RA. Moreover, we could not assess if absence of IGF-I may accelerate local or systemic bone damage as IGF-I knockout mice have a perinatal lethality of 95% [23]. Finally, we used here a model where IGF-I is expressed under the rat collagen type 1 alpha I promoter in osteoblasts, implicating that IGF-I will be strongly overexpressed upon activation of osteoblasts. We chose to use this model rather than a constitutive overexpression model as it allows to study the role of IGF-I more specifically and physiologically during active bone turn-over. At young age Ob-IGF-I^+/-^ males and females from the 324–7 line have elevated serum IGF-I levels and a concomitant increase in bodyweight during growth and development [19]. In the present CIA study, the Ob-IGF-I^+/-^ males show a significant increase in bodyweight compared to littermate controls (data not shown) however the mice, fully grown adults, show no difference in systemic levels of IGF-I compared to littermate controls (data not shown).

Therefore, we next hypothesized that IGF-I overexpression might enhance ongoing osteoblast-mediated syndesmophyte formation in SpA-like disease. To test this hypothesis we performed an experiment using the same transgenic mouse strain in the DBA/1 aging model for ankylosing enthesitis, where there is clear evidence of osteoblastic activity in the affected entheses and joints [22]. Aging males from different litters were mixed and housed together to induce continuous stress resulting in the spontaneous development of inflammation and ankylosis in the peripheral joints. Again IGF-I transgenic mice show a significant increase in bodyweight compared to wildtype littermates (data not shown). Clinically we could not observe differences between the two groups. Histologically we could not detect enhanced presence of syndesmophytes as indicated by equal presence of hypertrophic chondrocytes and osteoblasts in the Ob-IGF-I^+/-^ mice and non-transgenic littermates. We can conclude that IGF-I overexpression in the osteoblast lineage did not contribute to enhanced syndesmophyte formation in SpA-like disease.

We cannot exclude that the overexpression of IGF-I in both disease models could, on top of the phenotype of the IGF-I transgenic mice [19], could have subtle changes in bone and bone density as there were no histomorphometric analyses performed. We were specifically interested in pathological processes induced locally and concerning the remodeling of bone during inflammatory arthritis, either repair of erosions in RA or syndesmophyte formation in SpA. For these analyses semi-quantitative scoring is sufficient in our experience.

The fact that transgenic mice in both experiments had higher bodyweight compared to littermate controls indicates that the transgene was indeed operative. However we can obviously not exclude that forced constitutive overexpression of IGF-I may affect bone metabolism in general and new bone formation in particular, we chose to focus on the local mode of action as we were specifically interested in inflammation induced pathology occurring in the joints. However our data indicate that the activation of osteoblasts and therefore the IGF-I axis in both a destructive arthritis model and a remodeling ethesitis model is insufficient to promote either repair of erosions or pathological syndesmophyte formation.

In conclusion, our experiments clearly and consistently indicate that IGF-I overexpression in the osteoblast lineage does not contribute to enhancement of repair of bone erosions or syndesmophyte formation during experimental arthritis, although IGF-I has clearly a role in bone metabolism, our data concord to indicate that it is not a major determinant of osteoproliferation in inflammatory arthritis. It needs to be emphasized, however, that a lot of other factors are involved in the tightly regulated process of bone remodeling [3, 4, 8, 9]. We can thus not exclude that, although IGF-I overexpression on its own has no effect on osteoproliferation, it may enhance formation of new bone induced by other factors. Further research should identify other growth factors and molecular pathways playing a more central role in the pathophysiology of new bone formation during inflammatory arthritis.
